# Slag Formation during Reduction of Iron Oxide Using Hydrogen Plasma Smelting Reduction

**DOI:** 10.3390/ma13040935

**Published:** 2020-02-20

**Authors:** Masab Naseri Seftejani, Johannes Schenk, Daniel Spreitzer, Michael Andreas Zarl

**Affiliations:** 1 Department of Metallurgy, Montanuniversitaet Leoben, 8700 Leoben, Austria; Johannes.Schenk@unileoben.ac.at (J.S.); Daniel.Spreitzer@unileoben.ac.at (D.S.); Michael-Andreas.Zarl@unileoben.ac.at (M.A.Z.); 2 K1-MET GmbH, Stahlstraße 14, A-4020 Linz, Austria

**Keywords:** slag, basicity, hydrogen plasma, smelting reduction, iron oxide, plasma arc, hydrogen utilisation, degree of reduction, hematite

## Abstract

Replacing carbon by hydrogen is a huge step towards reducing CO_2_ emissions in the iron- and steel-making industry. The reduction of iron oxides using hydrogen plasma smelting reduction as an alternative to conventional steel-making routes has been studied at Montanuniversitaet Leoben, Austria. The aim of this work was to study the slag formation during the reduction process and the reduction behaviour of iron oxides. Furthermore the reduction behaviour of iron ore during continuous feeding was assessed. Mixtures of iron ore and calcined lime with a basicity of 0, 0.8, 1.6, 2.3, and 2.9 were melted and reduced by hydrogen. The off-gas composition was measured during the operations to calculate the process parameters. The reduction parameters, namely the degree of reduction, degree of hydrogen utilisation, produced iron, and slag, are presented. The results of the batch-charged experiments showed that at the beginning of the reduction process, the degree of hydrogen utilisation was high, and then, it decreased over the operation time. In contrast, during the continuous-feeding experiment, the degree of hydrogen utilisation could be kept approximately constant. The highest degrees of reduction and hydrogen utilisation were obtained upon the application of a slag with a basicity of 2.3. The experiment showed that upon the continuous feeding of iron ore, the best conditions for the reduction process using hydrogen could be applied.

## 1. Introduction

Hydrogen plasma smelting reduction (HPSR) was introduced at the Chair of Ferrous Metallurgy of the Montanuniversitaet Leoben in 1992. At the beginning, HPSR was part of the Ultra Low CO_2_ Steelmaking (ULCOS) programme. ULCOS was a European Union programme to find innovative solutions to reduce CO_2_ emissions in the steel industry. However, currently, HPSR is studied within the framework of the SuSteel project in Austria.

CO_2_ emissions have serious consequences on the environment. CO_2_ emissions comprise 65% of total greenhouse gas emissions [1]. The industry sector produced approximately 15.4 Gt of CO_2_-equivalent and 13.14 Gt of CO_2_ in 2010 [2]. CO_2_-equivalent is the conversion factor of greenhouse gases in terms of the warming potential relative to that of carbon dioxide. The iron and steel industry is responsible for 4%–7% [3] of the total greenhouse gases. The production of 1 ton of steel generated 1.83 tons of CO_2_ on average in 2017 [4].

**Figure 1:**
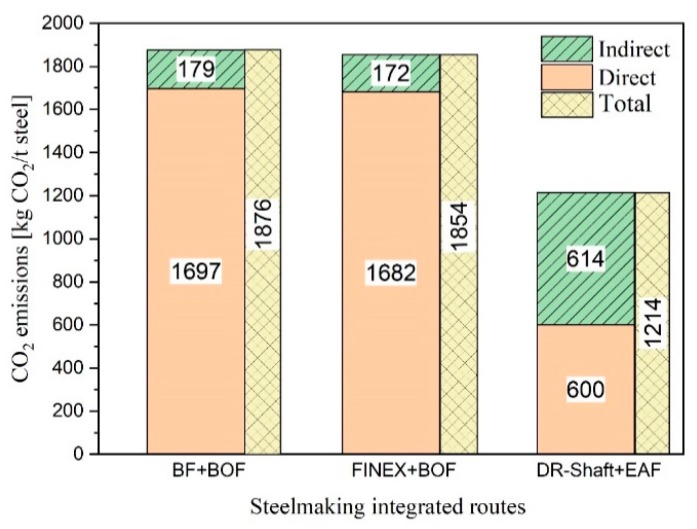
CO_2_ emission intensity of integrated steel-making processes [6,7].

Liesienko et al. [5] characterised various iron- and steel-making processes according to their CO_2_ emission. The integrated Hyl 3 Energiron electric arc furnace process route generates 1171 kg of CO_2_ emissions per ton of steel, which is the minimum among all the steel-making processes. Figure 1 shows the different iron- and steel-making process routes with their optimal amount of CO_2_ emissions [6,7].

CO_2_ emissions can be divided into two types: direct and indirect emissions. The former are emissions which occur directly at the production site, whereas the latter are the emissions from raw material transportation and the production of electrical energy and oxygen. For the conversion of the electrical energy production into CO_2_ emissions in the DR-shaft-EAF route, 1 kWh of energy is considered to be equal to 0.6 kg of CO_2_.

The integrated blast furnace-basic oxygen furnace route accounts for 71% of the total steel production, translating into 1876 kg of CO_2_/ton of liquid steel [7]. Compared with this, the integrated direct reduction shaft-electric arc furnace process generates less CO_2_ emissions. Therefore, the use of hydrogen as a reducing agent for CO_2_-free steel-making could be an appropriate alternative process [8,9].

With respect to the hydrogen species in the plasma state, they can exist as molecular $H_{2}$, atomic H, ionic $H^{+},H_{2}^{+},H_{3}^{+},$ and exited H* states. At ambient temperature, hydrogen is stable in its molecular H_2_ form. According to the Saha equation, hydrogen molecules start to dissociate at temperatures above 3000 K. The dissociation and ionisation of hydrogen atoms are two separate processes. At high densities and temperatures, hydrogen is ionised easily [10,11,12]. Hydrogen particles in the plasma state are strong reducing agents for iron oxides [13]. In the HPSR process, hydrogen in the plasma arc zone can be activated to be used for the reduction of iron oxides.

One of the main advantages of using HPSR is its ability to produce steel from iron ore powders without pre-agglomeration by using only a single-process step [14]. Numerous investigations have been carried out to produce metal from its ores by using hydrogen plasma [15,16,17,18,19,20,21,22]. They studied the reduction behaviour and the relevant parameters during the reduction of oxides using hydrogen in plasma state. The reduction of iron ore with hydrogen in a DC plasma jet was studied by Gilles and Clump [23]. They used a DC plasma jet with a water-cooled plasma torch to generate an arc for the smelting process. Iron ore powder with two different grain size distributions, 63–74 and 44–53 µm, was used to carry out the experiments. The iron ore powder was fed continuously to a water-cooled copper crucible in the reduction zone and was reduced using a flow of pure hydrogen or a mixture of argon and hydrogen in the plasma arc zone. They reported that the degree of reduction is decreased by an increase in the iron ore particle size. Moreover, the degree of reduction increases upon an increase in the plasma energy and the plasma temperature. Therefore, the heat transfer to oxide particles is the limiting kinetic factor for the reduction process.

The reduction behaviour during HPSR has been investigated by some researchers at Montanuniversitaet Leoben [15,24,25,26,27]. Badr et al. [8] studied the main parameters influencing the reduction behaviour of different iron ores in the HPSR process. One of the main results was a lower degree of hydrogen utilisation ($\eta_{H_{2}}$) when using a high concentration of hydrogen in the mixture of H_2_–Ar. Their results were in good agreement with the observations of other researchers [28,29,30,31,32]. In the present study, the concentration of hydrogen in the gas mixture was set to be constant.

The temperature and the basicity of the slag are parameters which influence the reduction behaviour, because partial solidification limits the transport of oxygen to the reduction interface. Badr [15] studied the ternary slag systems Al_2_O_3_–SiO_2_–FeO with the addition of 0, 10, and 20 wt.% CaO. He reported that the slag of Carajas ore with an FeO content of 30 wt.%, Al_2_O_3_ at 40 wt.%, and 0 wt.% CaO, at the temperature of 1500 °C, solidifies during the process. However, with an increase in the CaO content to 20 wt.% and at the basicity of 1, the slag is in the liquid state, which enables a 98% degree of reduction. An increase in the CaO content in the slag leads to an increase in the slag amount and the energy demand. Moreover, acidic slags decrease the lifetime of the refractories. Therefore, it was suggested that the process be operated at temperatures above 1600 °C and at a lower basicity. He also conducted some experiments with a basicity of 0.06, 1, 1.5, 2, and 2.5 to assess the effect of basicity on the dephosphorisation behaviour in the HPSR process. He reported that in the case of a higher basicity (B_2_: 1.5, 2, and 2.5), the phosphorous level decreased.

## 2. Materials and Methods

Although the equipment, materials, and methodology of the experiments were described in detail in the previous work [33], in this section, the main points, the new methods, and experiments are briefly described.

### 2.1. HPSR Laboratory Equipment

**Figure 2:**
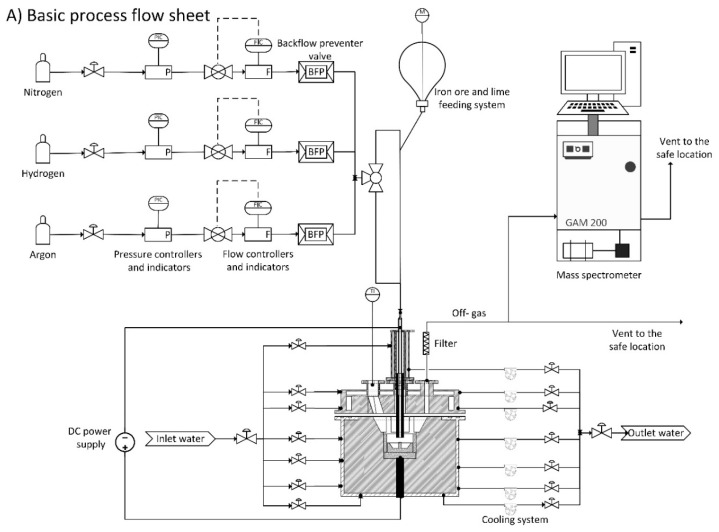

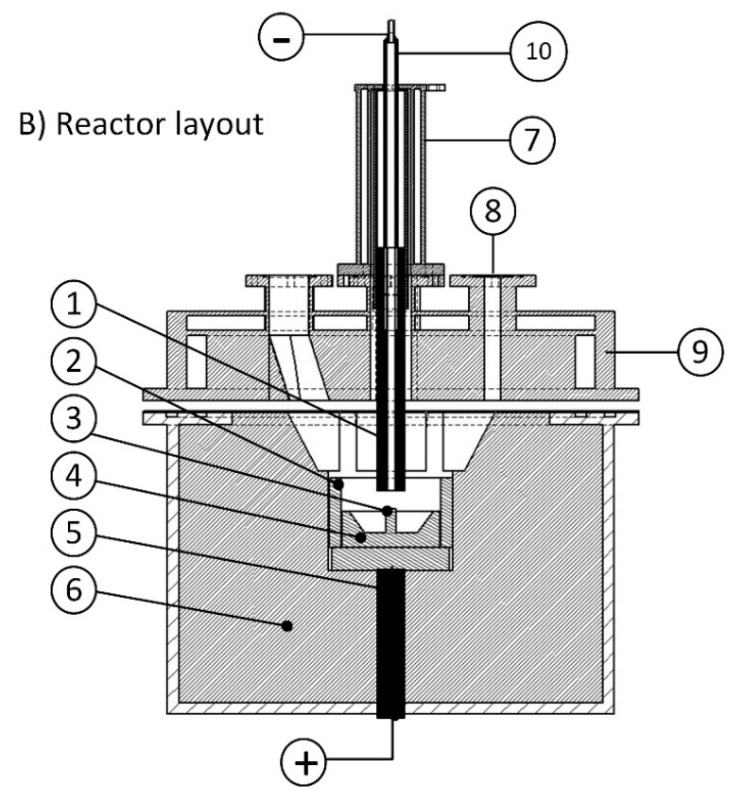
Process flow sheet of laboratory equipment: **A**) process flow diagram and **B**) reactor layout, reproduced from Reference [13] with permission. 1. Hollow graphite electrode (HGE); 2. Steel ring; 3. Ignition pin; 4. Steel crucible; 5. Bottom electrode; 6. Refractories; 7. Electrode holder with cooling system; 8. Four orifices to (a) install off-gas duct, (b) monitor the arc, (c) install a pressure gauge, and (d) install a lateral hydrogen lance; 9. Reactor roof with refractories and cooling cooper pipes; 10. Steel pipe to inject gases and continuous feeding of fine ores.

Figure 2 shows the process flow sheet of the hydrogen thermal plasma facilities in the laboratory of the Chair of Ferrous Metallurgy at Montanuniversitaet Leoben (Leoben, Austria), including the laboratory plant and the detailed reactor layout.

A DC electrical power supply with the maximum power of 12 kW (20–100 V and 80–150 A) was used to generate an arc between a hollow graphite electrode (inner diameter: 5–8 mm and outer diameter: 26 mm) and an ignition pin. The ignition pin with a diameter of 10 mm was welded at the centre of the steel crucible with a maximum capacity of 100 g of iron ore. A mixture of argon and hydrogen was injected through the hollow graphite electrode (HGE) during the operation to produce the ionised particles in the plasma arc zone. In a series of experiments, a premixed iron ore and lime was charged into a steel crucible before the run of experiments. The steel crucible was located inside a steel ring. Its inner side was covered by a refractory lining to protect the inner ring wall from the heat of the arc radiation.

**Figure 3:**
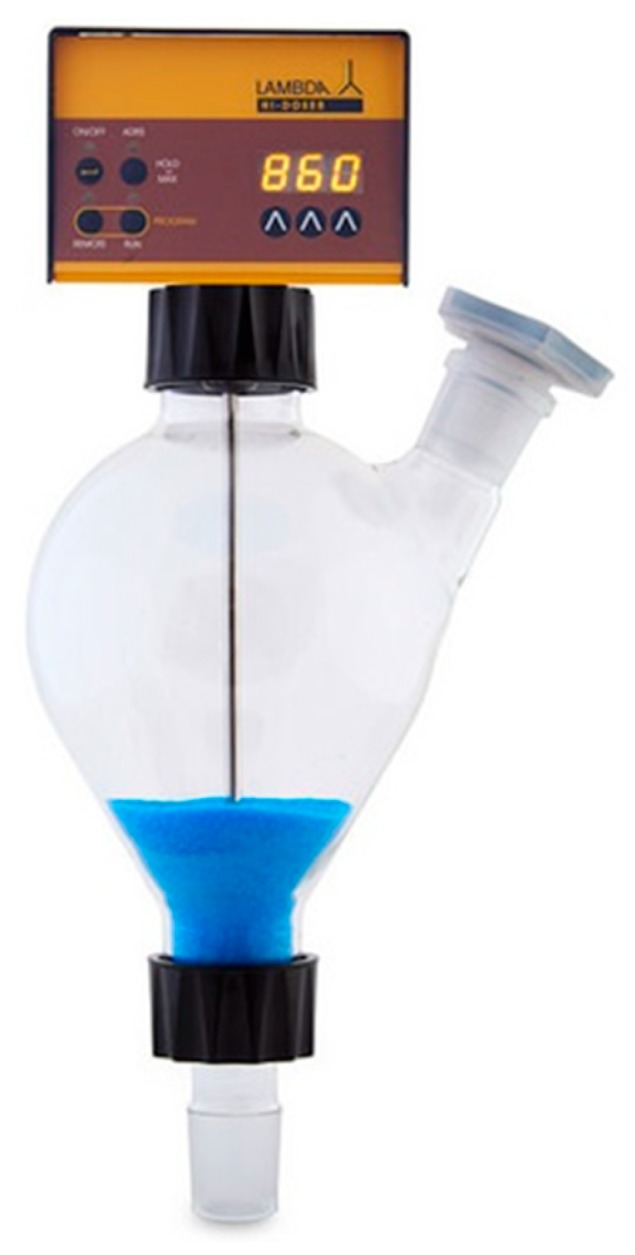
Hi-Doser 1 L, powder dosing instrument.

To conduct another experiment for this research work, a solid powder feeding system was used to feed the premixed iron ore and lime continuously into the plasma arc zone during the arc operation. Figure 3 shows the Hi-Doser 1 L powder dosing instrument produced by Lambda Laboratory Instruments (Brno, Czech Republic).

The powder dispenser consists of a dosing unit and a powder distributor coupled to a stepping motor to control the number of turns per minute and accordingly the dispensing rate.

A mass spectrometer (MS), GAM 200 (InProcess Instruments Gesellschaft für Prozessanalytik mbH, Bremen, Germany), was used to analyse the chemical composition of the off-gas. However, water vapour and dust were removed from the off-gas in the off-gas cleaning system before the off-gas entered the MS.

The optical emission spectrometer of the type Spectrolab (SPECTRO Analytical Instruments GmbH, Kleve, Germany) was used for the chemical analysis of the steel crucible, ignition pin, and the produced iron. Moreover, the optical microscope Nikon MM 40 (Nikon Corporation, Shinagawa, Tokyo,) with the image analysis software CLEMEX Vision 7 (CLEMEX, Longueuil, Quebec, J4G 1T5, Canada) was used for the study of the microstructure of the produced iron and to define the melted section of the crucible during smelting reduction. The FEI Quanta 200 Mk2 scanning electron microscope (SEM) equipped with an EDX device (Thermo Fisher Scientific, Waltham, Massachusetts, U.S.)) in the low vacuum mode was used to study the topographical and chemical analyses of the slags.

### 2.2. Experimental Programme and Materials

In the present study, two series of experiments were designed to study the slag formation and reduction behaviour of iron ore by using the HPSR process. Table 1 shows the experimental programme for the current study.

Five experiments were carried out with the basicity of 0, 0.8, 1.6, 2.3, and 2.9 by the addition of lime to iron ore. For instance, in Ex. 1, pure iron ore was used without any addition of lime, and 1.7 g of lime was added to 98.3 g iron ore in Ex. 2 to achieve a slag with a basicity of 0.8. One experiment with the basicity of 1.6 was carried out with a continuous feed of the powder into the crucible to evaluate the reduction behaviour.

To run the set of basicity experiments, 100 g of the premixed iron ore and lime was charged to the steel crucible before running the experiments. However, for the experimental run with continuous feeding, 20 g of the material was charged into the crucible and 80 g was continuously fed during the reduction process in 1380 s.

The flow rate of argon and hydrogen and the total flow rate for all the experiments were kept constant. The flow rate of the injected premixed argon and hydrogen was 5 L/min with the ratio of 50% Ar–50% H_2_. As in the previous study [33], hematite iron ore from the Carajas mine (Vale S.A., Parauapebas-State of Pará, Brazil) was used to run the experiments with the composition listed in Table 2.

The limestone was calcined at 1100 °C and then mixed with the iron ore powder. The chemical composition of the calcined lime is shown in Table 3.

The grain size distributions of iron ore and lime, which were separately classified, and then were mixed, are shown in Table 4.

During the experiment, the ignition pin and a part of the steel crucible were melted. The alloyed carbon from this melted steel material contributed to the reduction of iron oxides. Therefore, the compositions of these parts were important to consider in the mass balance. The chemical compositions of the steel crucible and the ignition pin are shown in Table 5.

### 2.3. Description of the Operation

The set of basicity experiments were conducted using the charge of 100 g of premixed powder into the steel crucible. Then, the plasma reactor was assembled, and oxygen was purged out from the system by an argon stream of 5 L/min. This was done until the oxygen amount shown by the MS reached less than 1%. Thereafter, the operation was started by the generation of the arc between the HGE and the ignition pin.

The first operational step was pre-melting. This was performed for 180 s to create a liquid pool of iron oxide. The pre-melting step was carried out under a pure argon flow at a rate of 5 L/min. The arc length at the start of the operation, which was the distance between the tip of the electrode and the ignition pin, was approximately 20 mm. After the meltdown of the ignition pin, the arc length became approximately 35 mm.

The next step was the reduction process, which was started by switching the gas from pure argon to a mixture of 50% argon–50% hydrogen at a total flow rate of 5 L/min. The operation time of Ex. 3 was 25 min. With an increase in the operation time, the transformer of the power supply got hot. Therefore, when the system was optimised, the operation time of the other experiments was extended. To achieve a high degree of reduction and to study the reduction behaviour, the operation time of three experiments was extended to 33 min, and two other experiments were completed in 37 min. After the arcing was stopped, the remaining hydrogen was removed from the system by purging with 5 L/min of nitrogen for 10 min. The chemical composition of the off-gas was recorded by using MS during operation.

**Figure 4:**
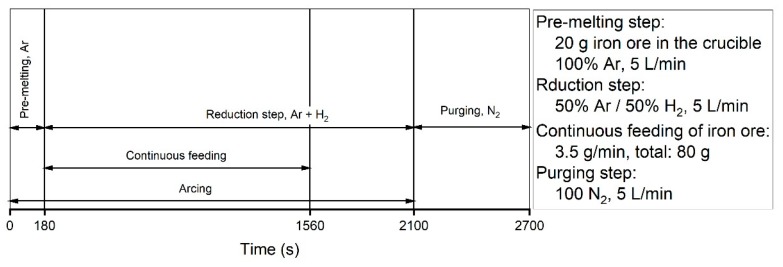
Process steps of Ex. 6 with the relevant parameters.

For conducting the continuous feeding experiment, 20 g of the premixed material was charged to the steel crucible and 80 g was charged to the glass vessel of the powder dosing instrument to be fed continuously and uniformly during the reduction step. The process steps of this experiment are shown in Figure 4.

The time of the pre-melting step was 180 s, and then, the reduction process was started with the start of the continuous feeding of the iron ore powder. The powder was continuously fed to the crucible for 1380 s at a feeding rate of 3.5 g/min. To compare the results of the continuous feeding experiment with those of the basicity experiments, the flow rate and the composition of the gas during the operations were the same for both series of experiments.

### 2.4. Methods

**Figure 5:**
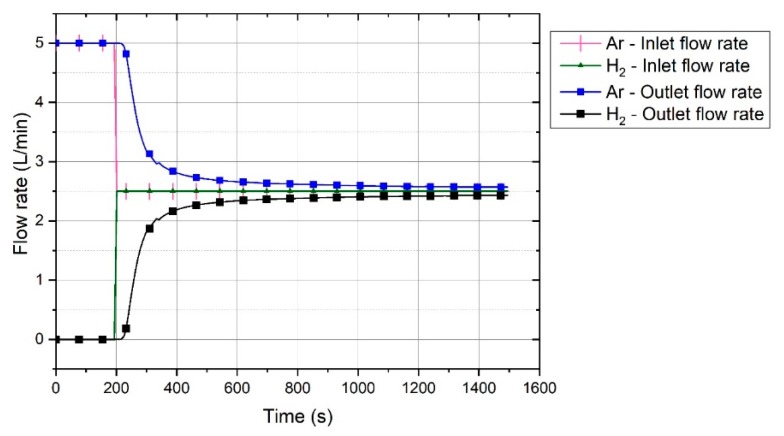
Reference diagram of inlet and outlet flow patterns of argon and hydrogen [33].

In the pre-melting step, only argon was injected to the reactor; therefore, the outlet gas consisted of Ar, CO, and CO_2_. With the start of hydrogen injection to the system, the outlet gas included Ar, CO, CO_2_, H_2_, and H_2_O, where CO, CO_2_, and H_2_O were the reduction products. Taking a closer look inside the reactor, you need to keep in mind that the gas is not a uniform mixture, because the inserted hydrogen needs some time to be distributed uniformly, and while this is happening, hydrogen is also taking part in the reduction process. To calculate the outlet flow rate of each gas, a reference diagram was prepared with an injection of a 5-L/min flow consisting of a 50% Ar–50% H_2_ gas without arcing to a prefilled reactor with argon. Figure 5 shows the reference diagram, which illustrates the inlet and the outlet flow rate of hydrogen and argon over the time.

The flow pattern of hydrogen and argon was used as a reference diagram to calculate the flow of each gas for every experiment. The calculation procedure is explained in detail in Reference [33]. The reference diagram shows the trends of the outlet flow rate of hydrogen and argon. Upon the start of hydrogen injection, the two gases showed the set flow rate only after a period of time.

Because of the reduction reaction, a partial molar volume of hydrogen reduced iron oxides and produced water. Water vapour was condensed and separated from the gas in the off-gas cleaning system. Therefore, the amount of produced water was the difference between the amount of hydrogen shown by MS and the amount of hydrogen from the reference diagram. The hydrogen utilisation degree $\eta_{H_{2}}$ was calculated as follows:

(1) $$\eta_{H_{2}}=\frac{{\%H}_{2}O}{\left({\%H}_{2}+{\%H}_{2}O\right)}\times 100$$

Iron oxide was reduced by hydrogen or carbon, when the latter was introduced from the steel crucible, ignition pin, or HGE. Therefore, not only hydrogen but also carbon contributed to the reduction reactions. The degree of reduction by hydrogen (R_D,H2_), that by carbon (R_D,C_), and the total degree of reduction by hydrogen and carbon (R_D,H2+C_) were calculated as follows:

(2) $$R_{D,H_{2}}=m_{O,H_{2}O}/m_{O,\mathrm{in}\;\mathrm{iron}\;\mathrm{oxide}\;}\times 100$$

(3) $$R_{D,C}=(m_{O,\mathrm{CO}}+m_{O,{\mathrm{CO}}_{2}})/m_{O,\mathrm{in}\;\mathrm{iron}\;\mathrm{oxide}}\times 100$$

(4) $$R_{D,H_{2}+C}=R_{D,H_{2}}+R_{D,C}$$

where $m_{O,H_{2}O}$, $m_{O,\mathrm{CO}}$, $m_{O,{\mathrm{CO}}_{2}}$, and $m_{O,\mathrm{in}\;\mathrm{iron}\;\mathrm{oxide}}$ are the masses of oxygen in H_2_O, CO, CO_2_, and iron oxide, respectively. Please note that all the reduction parameters were calculated using the chemical composition of the off-gas. However, to define the carbon source, a mass balance was performed. For the performance of the mass balance, the electrode, steel crucible, and the ignition pin were weighed before and after each test run. The weight changes of the HGE before and after the experiments showed the amount of carbon introduced into the reduction reactions. The steel crucible was partially melted during the operation, and the carbon from the crucible contributed to the reduction reactions. Therefore, to estimate the amount of carbon from the steel crucible introduced into the melt of the iron oxide, the steel crucible was cut in half, and the macro- and microstructure of the cross-section were observed with an optical microscope to estimate the amount of the melted section of the crucible. Consequently, the amount of carbon was calculated for each test run by using the partially melted section of the crucible and the carbon content of the steel.

The reduction rate by hydrogen was the rate of oxygen removed from the iron oxide upon the formation of H_2_O. It was calculated as follows:

(5) $$\mathrm{Reduction}\;\mathrm{rate}={\mathrm{dm}}_{O,H2O\;}\left(g\right)/\mathrm{dt}\;\left(\min\right)$$

The produced iron was obtained by the calculation of the mass balance. Accordingly, the degree of metallisation (M_D_), which is the ratio of metallic iron to total iron in iron ore, was calculated as follows:

(6) MD=Produced iron (g)Total Fe (g)×100

Furthermore, M_D_ was calculated as a function of the degree of reduction as follows:

(7) $$M_{D}=\frac{R_{D}-33.3}{66.6}\times 100$$

## 3. Results and Discussion

Two sets of experiments were conducted to study the reduction behaviour of iron oxide and slag formation. For this purpose, the off-gas composition, produced iron, slag, R_D_, $\eta_{H_{2}}$, and reduction rate were studied.

### 3.1. Off-Gas and Produced Iron

**Figure 6:**
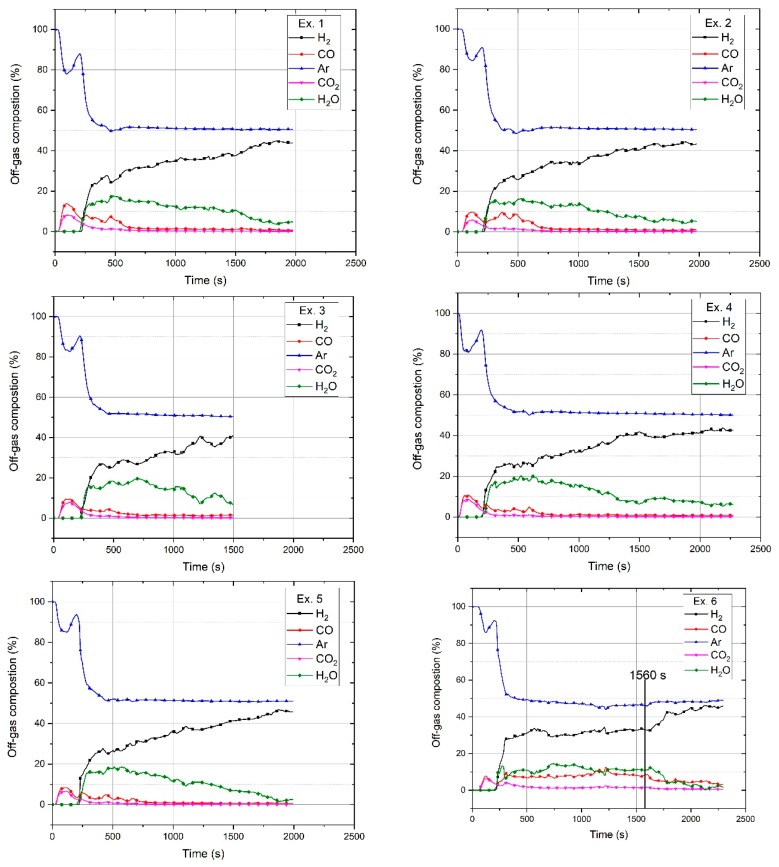
Chemical composition of off-gas for Exs. **1**, **2**, **3**, **4**, **5**, and **6**.

Argon as an inert gas left the reactor without any reaction, and hydrogen reacted with the iron oxides, namely Fe_2_O_3_, Fe_3_O_4_, and FeO, to form H_2_O. The off-gas composition during the pre-melting step consisted of Ar, CO, and CO_2_, and in the reduction step, H_2_, Ar, CO, CO_2_, and H_2_O. The produced water vapour was condensed in the off-gas cleaning system and was therefore not measured by the MS. Hence, the amount of water vapour was calculated and added to the composition graph of each test run. Every mole of hydrogen produced one mole of water; therefore, the amount of produced water vapour was the difference between the outlet hydrogen shown by MS and the reference diagram within a certain time period. The chemical compositions of the off-gas for the six experiments are shown in Figure 6.

Before starting the test runs, all the oxygen in the system was purged out by the injection of argon into the reactor. Therefore, at the start of the pre-melting step, only argon was inside the reactor. Upon the start of arcing and during the pre-melting step, 5 L/min of argon was injected into the reactor for 180 s. Carbon from HGE was introduced into the reduction reactions. Carbon reduced iron oxide and formed CO and CO_2_. A possible reason for the introduction of a high amount of carbon into the melt at the beginning of the process was that the HGE was cold and a thermal shock could cause a high erosion of the HGE and create a peak of CO and CO_2_ in the pre-melting step. With the formation of CO and CO_2_, the percentage of argon decreased. Then, the reduction process was carried out with an injection of a mixture containing 50% Ar–50% H_2_ at a total flow rate of 5 L/min. The trends of the gas compositions of all the experiments were similar except for Ex. 6, in which iron oxide was continuously charged into the reactor. The amount of H_2_O at the beginning of the experiments was high, and then, it decreased because of a decrease in the reduction rate. Ex. 6 had a different trend in terms of the H_2_ and H_2_O amounts, which is discussed in Section 3.2. The CO and CO_2_ produced by Ex. 6 was higher than that of the other experiments; the reasons for this are discussed at the end of this section. At 1560 s, continuous feeding was stopped, and the amount of CO and CO_2_ decreased.

Ex. 3 was stopped at 1500 s upon reaching the temperature limit of the transformer of the power supply. To achieve a high degree of reduction and to study the reduction behaviour, the system was optimised and the operation time of Exs. 1, 2 and 5 was extended to 1980 s; Exs. 4 and 6 were carried out in 2220 s.

**Figure 7:**
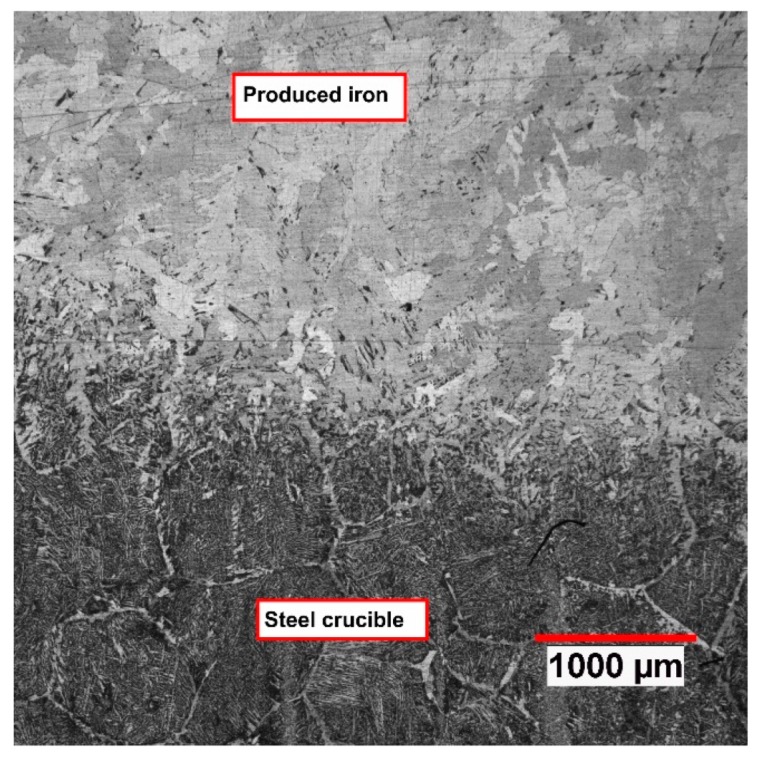
Cross-section of the crucible, partially melted crucible and mixed with produced iron, Ex. 3.

Carbon contributed to the reduction processes to reduce iron oxides from three sources, namely the ignition pin, steel crucible, and HGE. During the reduction process, the ignition pin was completely melted, and the steel crucible melted partially. The partially melted section of the crucible got mixed with the produced iron and the pin material. To calculate the amount of carbon that entered into the melt from the crucible, the microstructure of the middle cross-section of the crucible was assessed using an optical microscope. Figure 7 shows a cross-section of the crucible after the reduction operation.

Figure 7 shows the weld interface and the fusion depth. The ferritic microstructure of the fusion phase illustrates a low carbon content. Nevertheless, the chemical composition of the produced iron was analysed and is presented in Table 6.

The chemical composition shows that the carbon concentration is low; therefore, it means that carbon has reduced the iron oxide and formed CO or CO_2_. The melted section of the steel crucible for each test run was observed by optical microscopy, and then, the weight was estimated according to the fusion depth. Finally, the amount of carbon contributed to the reduction reactions by the steel crucible was calculated by the multiplication of the weight of the melted section and the carbon concentration. The other source of carbon was the HGE. Iron oxide fines during the continuous feeding stuck on the inner surface of the electrode and were reduced by carbon. This case was more obvious during the continuous feeding of iron ore. Moreover, the eroded particles from the HGE were introduced into the melt. Furthermore, the plasma arc caused the formation of iron oxide spatters, which stuck on the tip of the electrode and got reduced by the carbon material of the HGE.

In the pre-melting step, only argon was injected into the plasma reactor. Therefore, Ar, CO, and CO_2_ were the gaseous products. The amounts of produced CO and CO_2_ and their ratio were approximately in the same range for all the test runs except Ex. 6. For conducting Ex. 6, 20 g of the premixed powder was charged to the crucible; then, after switching the operation from the pre-melting to the reduction step, the continuous feeding of the powder was started. Therefore, the low amount of iron oxide inside the crucible led to a decrease in the CO and CO_2_ amounts at the beginning of the operation in comparison to the other experiments. However, the amounts of CO and CO_2_ emitted during the continuous feeding were higher than those of the other experiments. The possible reasons could be as follows:

1. sticking of fines on the inner surface of HGE and the direct reduction of hematite with carbon,
2. wear of the inner section of the HGE by the inflight particles of the powder, and/or
3. generation of more slag droplets on the tip of the electrode because of entering particles of iron ore to the melt.

It is clearly shown in Figure 6 that after continuous feeding was stopped at 1560 s, the amounts of CO and CO_2_ decreased. The total carbon in the off-gas was calculated using its chemical composition from MS. However, to define the carbon source, the mass balance of the HGE, ignition pin, and steel crucible was carried out. The HGE was weighed before and after each test run to calculate the loss of weight. Because the ignition pin was completely melted, the total carbon content was introduced into the melt. Table 7 shows the amount of carbon contributed to the reduction reactions by different sources for all the experiments (in grams).

The maximum amount of carbon from the HGE was contributed to the reductions observed in Ex. 6, in which the materials were continuously fed to the crucible. The melted section of the crucible differed from experiment to experiment.

### 3.2. Degree of Hydrogen Utilisation $\left(\eta_{H_{2}}\right)$

**Figure 8:**
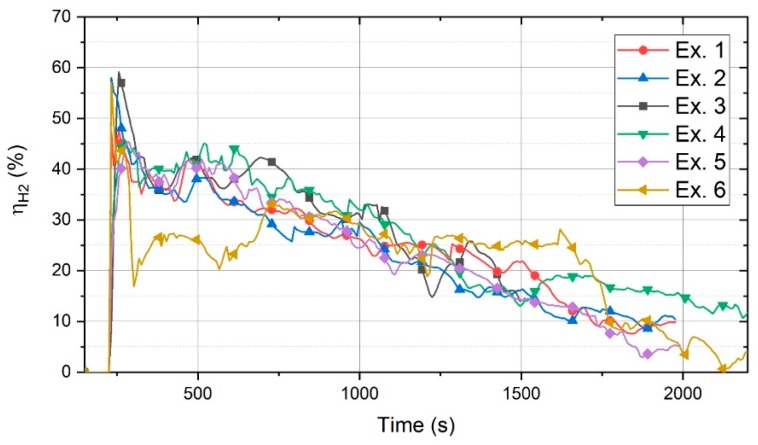
Degree of hydrogen utilisation $\eta_{H_{2}}$

The $\eta_{H_{2}}$ value was calculated by Equation (1) using the chemical composition of the off-gas and the reference diagram. The $\eta_{H_{2}}$ values for the six experiments are shown in Figure 8.

At the beginning of the reduction process (190–280 s) of Exs. 1 to 6, $\eta_{H_{2}}$ of all the experiments was high, and then, it decreased because a small amount of hydrogen was inside the reactor and there was a possibility of the reduction of a liquid pool of iron oxide. Therefore, a large portion of hydrogen could reduce iron oxide and form water. During the operation, iron oxide was reduced and its amount decreased, which caused a decrease in $\eta_{H_{2}}$. There was no considerable difference between the $\eta_{H_{2}}$ values of these experiments. However, it seems that Ex. 3 with a basicity of 2.9, the highest basicity, had the highest $\eta_{H_{2}}$ value most of the time. Please note that the generation of the arc between the HGE and the crucible, instead of between the HGE and the iron oxide, decreased the volume of the molten iron oxide, which led to a decrease in $\eta_{H_{2}}$.

Ex. 6 exhibited a different behaviour. First, as in the other experiments, $\eta_{H_{2}}$ was high, and then, after 230 s of operation, it decreased considerably. The reason could be the transfer of the arc toward the outer surface of the steel crucible, because the bath was at the usage of 20 g of ore, which was too small to keep the arc in place. Therefore, a large volume of the steel crucible was empty. Thereafter, $\eta_{H_{2}}$ gradually increased because of the continuous feeding of the material. During the continuous feeding, $\eta_{H_{2}}$ remained approximately constant until the continuous feeding of the ore was stopped (at 1560 s). After the termination of feeding, $\eta_{H_{2}}$ decreased significantly.

### 3.3. Degree of Reduction (R_D_)

**Figure 9:**
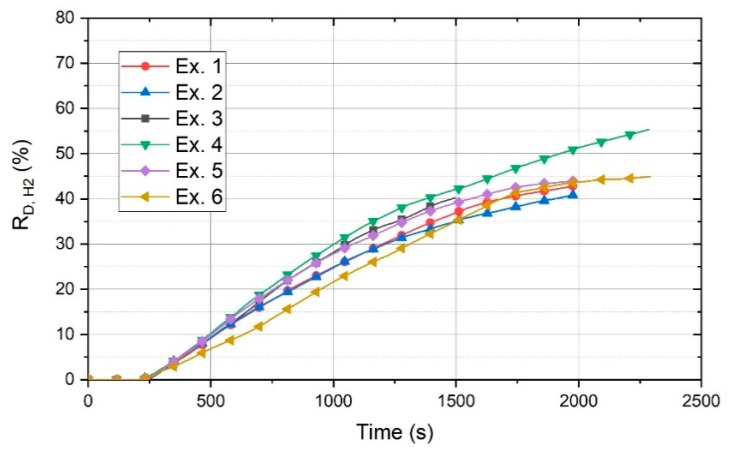
Degree of reduction by hydrogen (R_D,H2_).

The degree of reduction by H_2_ and H_2_/CO was studied, and the results are discussed in this section. Figure 9 shows the degree of reduction of 100 g of powder by hydrogen (R_D,H2_) for all the experiments.

The figure shows that the basicity of 2.3 is the best value to reach the highest R_D_. Exs. 1 and 2 with a basicity of 0 and 0.8, respectively, exhibited the minimum amount of R_D_. However, after 1250 s, Ex. 1 had better conditions in terms of R_D_. The R_D_ values of Exs. 3 and 5 with a basicity of 1.6 and 2.9, respectively, were approximately in the same range.

**Figure 10:**
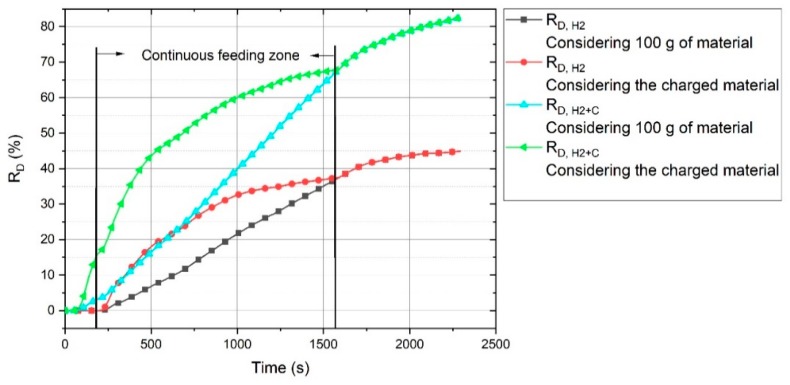
R_D,H2_ regarding the total material and the charged material of Ex. 6.

The R_D_ value of Ex. 6, regarding 100 g of the charged material, was in the average range of the other experiments. In this diagram, R_D_ for Ex. 6 was calculated on the basis of the total material (100 g) and not only the charged material, because for the batch-charged experiments, all of the materials were not in the liquid state during the operation. However, R_D_ for Ex. 6 gradually increased to reach the values of the other experiments by the passage of the operation time. The continuous material feeding provided better conditions for the reduction processes, because, for a batch charge, the arc could be generated between the HGE and on the surface of the previously reduced section. In contrast, the continuous material feeding provided iron oxides for the reduction. The real R_D,H2_ and R_D,H2+C_ values of Ex. 6 in consideration of the total and only the charged material were calculated, and the corresponding graph is shown in Figure 10.

The black line shows the R_D,H2_ value considering 100 g of the material. It shows that at the beginning of the reduction process, the real reduction degree was high, and then, upon the termination of continuous feeding, they became equal.

**Figure 11:**
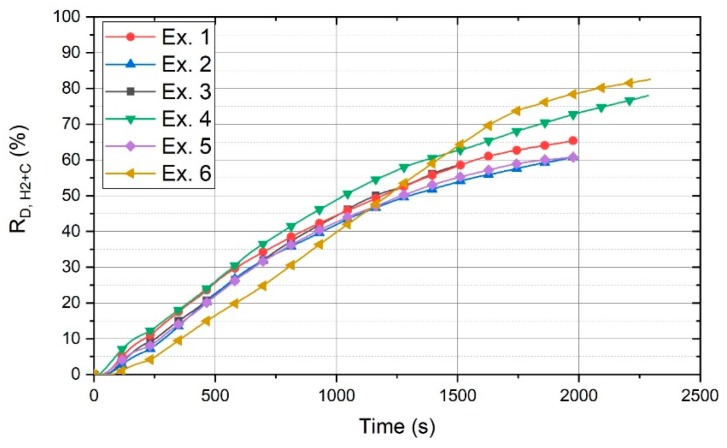
Total reduction degree, regarding the reduction by carbon and hydrogen.

The contribution of carbon to the reduction processes is shown in Figure 11. This figure shows the total degree of reduction (R_D,H2+C_) of each experiment, which is the sum of the degree of reduction by carbon (R_D,C_) and hydrogen (R_D,H2_).

The amount of carbon contributed to the reduction of iron oxides was approximately in the same range for the experiments with different basicities. Hence, the degree of reduction of each experiment increased uniformly. In contrast, the degree of reduction of Ex. 6 increased considerably. After 1500 s, the total degree of reduction of Ex. 6 was greater than that of the other experiments.

To rule out the contribution of carbon to the reduction reactions, a tungsten electrode could be used instead of the HGE. Badr [15] conducted a set of experiments by using a tungsten electrode to compare the reduction rate of hematite using hydrogen thermal plasma with HGE. He subtracted the oxygen reduced by carbon from the HGE and then reported that the reduction rates of iron oxide by a tungsten electrode and the HGE were in good agreement.

### 3.4. Reduction Rate

**Figure 12:**
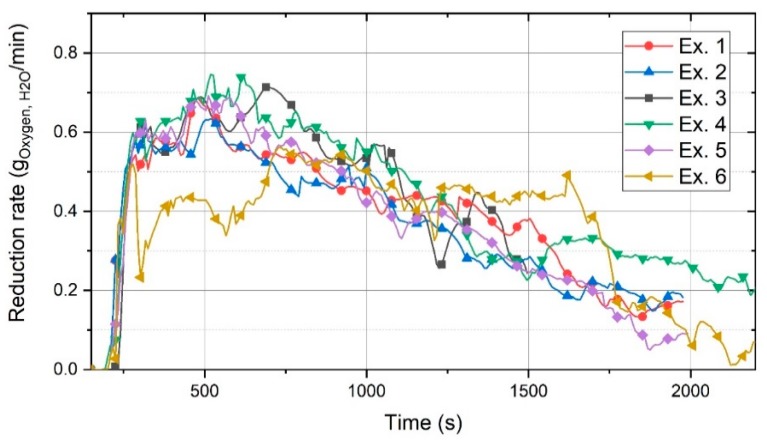
Reduction rate of iron oxides by hydrogen.

The reduction rate of iron oxide considering only hydrogen was calculated for each experiment, and the corresponding results are shown in Figure 12.

This graph shows the rate of oxygen removed by the formation of H_2_O. The changes and the reasons for these changes were similar to those for the graph of $\eta_{H_{2}}$ (Figure 8). Here, 0.73 g/min was the highest reduction rate, and upon the passage of time, it decreased to less than 0.2 g/min.

The reduction rate of Ex. 6 was lower than that of the other experiments in the time frame between 220 s and 750 s because of the low amount of iron oxide inside the crucible. However, the continuous charging of iron ore led to an increase in the reduction rate. Please note that the reduction rate was between 0.4 and 0.5 g/min in the time range of 1250–1700 s, which was higher than that of the other experiments. This illustrated that the reduction rate could be kept constant and optimised in the case of the continuous feeding of iron ore.

### 3.5. Produced Iron and Slag

The mass and energy balance were carried out to calculate the weight and the chemical composition of the slags. The chemical composition of each slag was calculated by using the R_D_ value of each experiment and the chemical composition of the samples (mixtures of iron ore and lime) and off-gas. Table 8 shows the values of the main parameters of the products and the co-products of each experiment for the operation time of 1495 s.

Hematite was reduced to Fe and FeO, wherein the former was considered to be the produced iron and the latter was a part of the slag. In 1495 s, the maximum amount of metallisation, which is the ratio of the produced iron to the total iron, was 46 wt.% for Ex. 4 with a basicity of 2.3, and the minimum metallisation belonged to Ex. 2 with a basicity of 0.8. However, the M_D_ value of Ex. 4 was calculated using Equation (7) as a function of R_D_, whose result was 44 wt.%. The calculation of M_D_ is theoretically correct if the iron oxide inside the slag would be only FeO and not Fe_2_O_3_. However, inside the steel crucible after each test run, there was no reduced or even melted ore. Hence, there was a deviation of less than 3% between the M_D_ values obtained by the two methods.

$\bar{\eta_{H_{2}}}$ of Ex. 6, which was 24.23 wt.%, was less than that of all the other experiments. Nevertheless, the metallisation was 47.64 wt.%, which was higher than that of all the other experiments.

To assess the efficiency of the process at longer times, the operation time of some experiments was extended up to 1975 s. Table 9 shows the calculated results of the main reduction parameters.

The produced iron and metallisation increased by an increase in the operation time. The difference between the metallisation of Ex. 6 in 1495 s and 1975 s was considerably higher than that of the other experiments because of the continuous feeding of the iron ore to the crucible, which caused the iron oxides in the reduction zone to be reduced. This interpretation can be exemplified for a low decrease in $\bar{\eta_{H_{2}}}$ for Ex. 6. This illustrated that in a continuous process of HPSR, the reduction rate can be kept constant.

R_D,H2_ in 1975 s was in every case more than 40%, while R_D,H2+C_ could reach 78% with the contribution of carbon to the reduction processes. The erosion rate of HGE and the amount of CO_2_-emissions per ton of the produced liquid metal were presented in the previous work [33].

**Figure 13:**
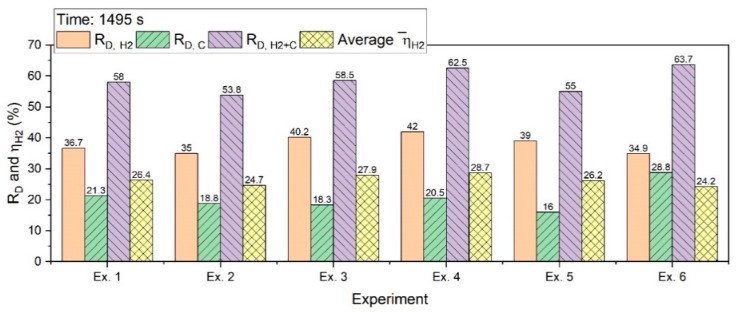
R_D_ and $\bar{\eta_{H_{2}}}$ in 1495 s

Figure 13 shows R_D,H2_, R_D,C_, R_D,H2+C_, and $\bar{\eta_{H_{2}}}$ for an operation of 1495 s.

R_D,C_ for the batch charging experiments was in the range between 15% and 22%; however, for the continuous feeding of iron ore, it was 28.8%. The reasons for this have already been explained in Section 3.1. R_D,H2+C_ was between 53% and 64%; Ex. 4 with a basicity of 2.3 had the highest degree among the batch experiments. A high contribution of carbon in the reduction process caused an increase in the total reduction degree for Ex. 6.

**Figure 14:**
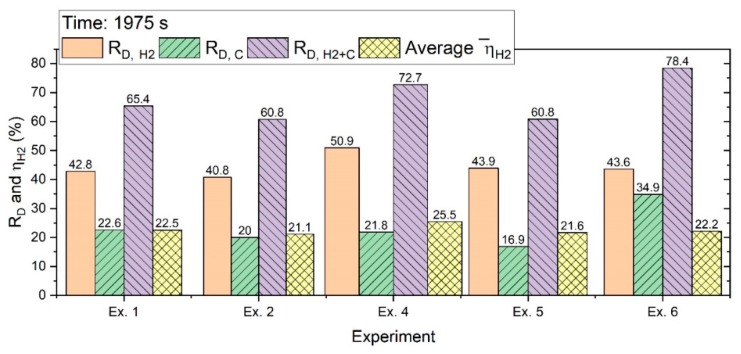
Degree of reduction and the average $\eta_{H_{2}}$ after 1975 s of operation.

The $\bar{\eta_{H_{2}}}$ value for the experiments of the batch charge in 1495 s was more than 25%; then, with the extension of the experiments, it decreased. Figure 14 shows the R_D_ value calculated in 1975 s.

With the extension of the experiments, the degrees of reduction increased. However, the $\bar{\eta_{H_{2}}}$ decreased because of a decrease in the reduction rate over the time of the operation.

### 3.6. Slag

The weight of the slag for each case is mentioned in Table 8 and Table 9. To calculate the chemical composition of the slag, mass and energy balance were carried out, as it was not possible by the calculations to define the amount of Fe_2_O_3_ and FeO because Fe could be present in the slag in both the forms. Therefore, the phase stability was studied by FactSage^TM^ 7.2 (Thermfact/CRCT (Montreal, Canada) at equilibrium in the same range of the reduction zone temperature. For instance, the slag composition of Ex. 5 was calculated for an operation time of 1975 s and is presented in Table 10.

**Figure 15:**
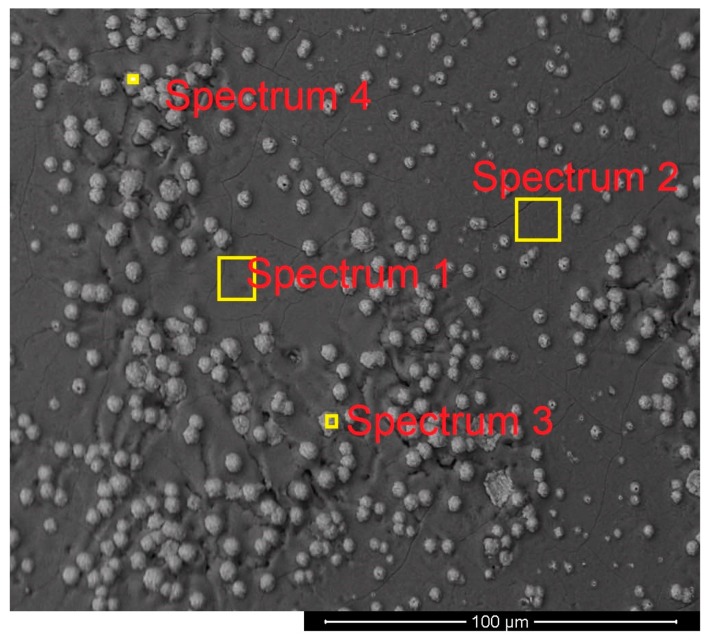
Morphology and the locations of spectra on the upper side of the slag layer of Ex. 5.

The composition and the morphology of the slags of Exs. 5 and 6 were studied by SEM. At the end of the experiments, a layer of slag covered the produced iron, and the morphology and the chemical compositions of both sides were studied. Figure 15 shows the morphology and the locations of the spectra on the upper side of the slag layer of Ex. 5.

It mainly consisted of white balls, which are iron ore particles, in a matrix of slag. The distribution of the ore particles was not uniform.

**Figure 16:**
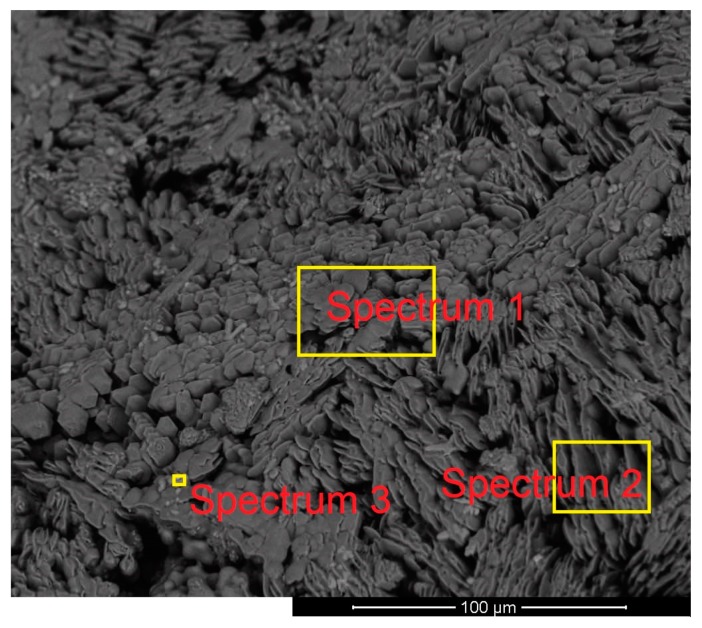
Morphology and the locations of spectra on the lower side of the slag layer of Ex. 5.

Furthermore, the lower side of the slag layer was assessed in terms of its morphology and chemical composition. Figure 16 shows the microstructure of the lower side of the slag with the locations of the spectra.

On the lower side of the slag, the iron oxide particles were not observed. This means that iron oxide particles were melted and mixed with slag before reaching the lower side of the slag.

**Figure 17:**
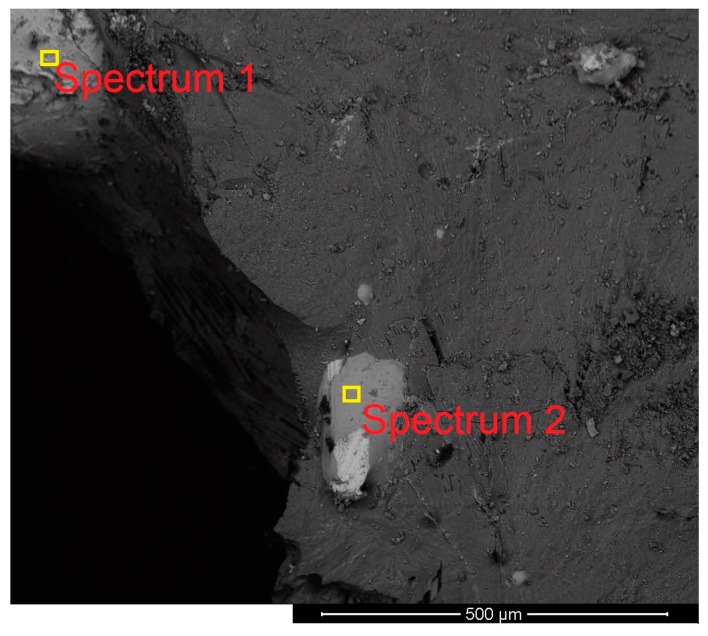
Section of slag with some droplets of produced iron for Ex. 6.

Figure 17 shows a section of slag of Ex. 6 with some droplets of the produced iron.

It shows that the iron oxide particles during continuous feeding were reduced and formed iron droplets in a matrix of slag. The droplets were grown to be heavier to leave the slag layer to penetrate into the melt.

The composition of the slags was measured at different locations by means of EDX, and the corresponding results are presented in Table 11.

Spectra 1 and 2 of Figure 15 and 1, 2, and 3 of Figure 16 show a difference between the composition of the lower side and the upper side. On the lower side, the amount of iron oxides was less than that of the upper side because of the reduction process. The amounts of Mg and Mn on the lower side were higher than those of the other side. To check the results of EDX, a sample of slag from Ex. 5 was analysed by using X-ray fluorescence (XRF), and the corresponding results are shown in Table 12.

Nevertheless, Fe could exist in the form of Fe^2+^ or Fe^3+^ in the slag; this is mentioned in the table as Fe^2+^ because by XRF, it was not possible to define the amounts of Fe^2+^ and Fe^3+^.

**Figure 18:**
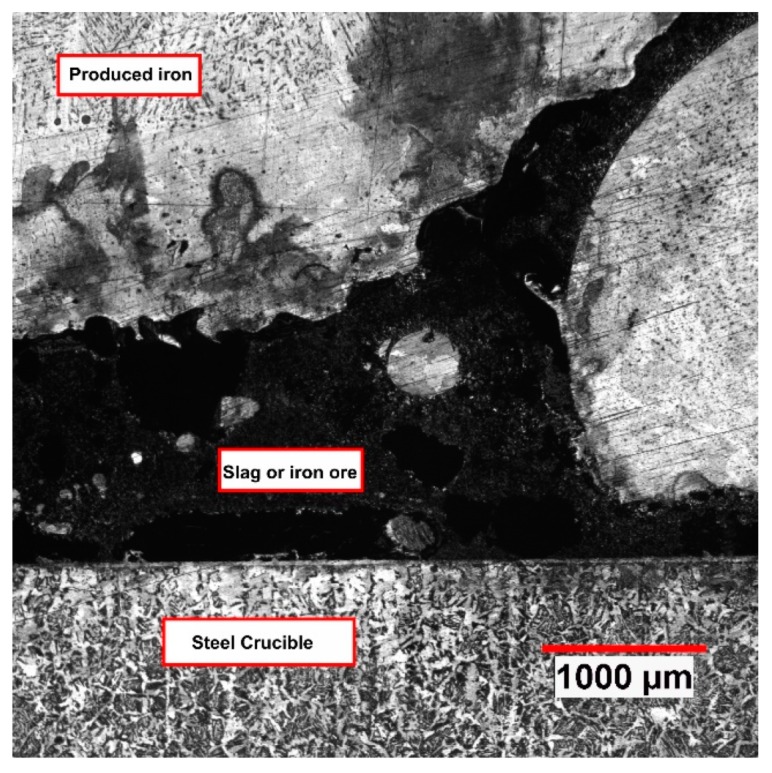
A cross-section of the crucible, unmelted crucible, a layer of slag or iron ore under a layer of produced iron, Ex. 2.

There was a large difference between the calculated chemical composition of the slag (Table 10) and the results of XRF (Table 12). The real composition of the slag showed that the basicity of the slag was lower than that expected. The basicity should have been 2.9; however, it was 1.6 according to Table 12. The main reason was the entry of the material from the refractory to the melt. Sodium silicate was used as a binder for the application of the refractory lining. Therefore, the melting of the refractory material and mixing with slags increased the amount of SiO_2_, which was why the real basicity was lower. The other point for the deviation of the real composition of the slag was the high reduction degree of iron oxide. Because slag was not homogenised, the iron oxide particles exposed to the reduction agent and plasma arc were reduced and the rest buried by the partially melted material in the steel crucible could not be reduced. This led to the production of a non-homogenised slag in the crucible. Therefore, to achieve the best conditions for the reduction process, the material inside the crucible should be completely melted. However, in the laboratory-scale facility, it was not possible to keep the material in the liquid phase during the operation, because of the use of a low-power furnace and cooling the system with water. Therefore, the achievement of a high degree of reduction was not possible with the laboratory-scale facility. Figure 18 shows the slag or iron oxide buried by the produced iron.

The figure shows a mass of slag or non-reduced iron oxide buried under a layer of produced iron. Therefore, even with the extension of the operation time, the chance of reducing this section of material was low.

The composition of the slag in Table 12 shows that the FeO content in the slag was less than 7%. This means that it was feasible to achieve a high degree of reduction in this process if the material could be melted and contacted with the reducing agent.

According to the composition of iron ore and lime, it was expected to produce a slag with a higher content of Al_2_O_3_ than that of MgO. However, due to the introduction of MgO from the refractory lining, the amount of MgO in the slag was higher than that of Al_2_O_3_. To analyse the liquidus temperature of the slag, two ternary systems, SiO_2_–FeO–Al_2_O_3_ and SiO_2_–FeO–MgO, with the additions of CaO were studied using FactSage^TM^ 7.2. The maximum calculated basicity was 2.9 for Ex. 5; however, the chemical composition of the slag showed that it decreased to 1.6 due to the mixing of the refractory lining material with the melt. During the reduction process, the CaO amount in the slag continuously increased due to the decrease in the iron oxide.

**Figure 19:**
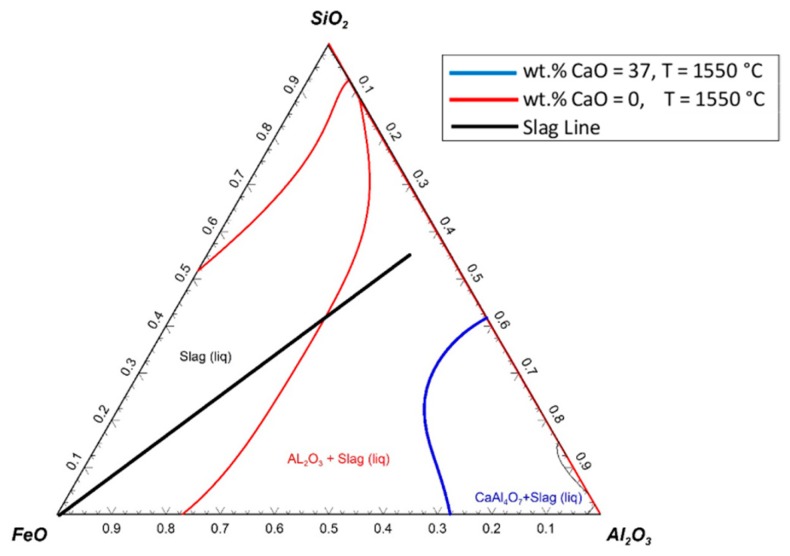
Ternary system of SiO_2_–FeO–Al_2_O_3_ with various addition of 0% and 37 wt.% CaO at 1550 °C.

A set of calculations was carried out to assess the possible solidifications of the SiO_2_–FeO–Al_2_O_3_ system for the slags with 0% and 37% of CaO at 1550 °C. The results are shown in Figure 19.

The presumptive slag line shows the changes in the slag composition during the reduction process. Nevertheless, the slag line was assumed to be a straight line; it could have different paths according to the melting and the reduction process. At the beginning of the reduction process, the FeO content in the slag was high, and then, because of the reduction process, it decreased. The red line shows the liquidus temperature of the slag without any addition of CaO. The solid particles could be formed if the FeO content decreased to less than 30%. The blue line shows the liquidus temperature of the slag with 37 wt.% CaO; therefore, the graph was normalised to 100% for the other components of the slag (SiO_2_–FeO–Al_2_O_3_). The slag line did not cross the line of the chart where 37 wt.% of CaO was added to the slag at 1550 °C; the slag always stayed in the liquid state. Hence, the reduction rate could be improved.

**Figure 20:**
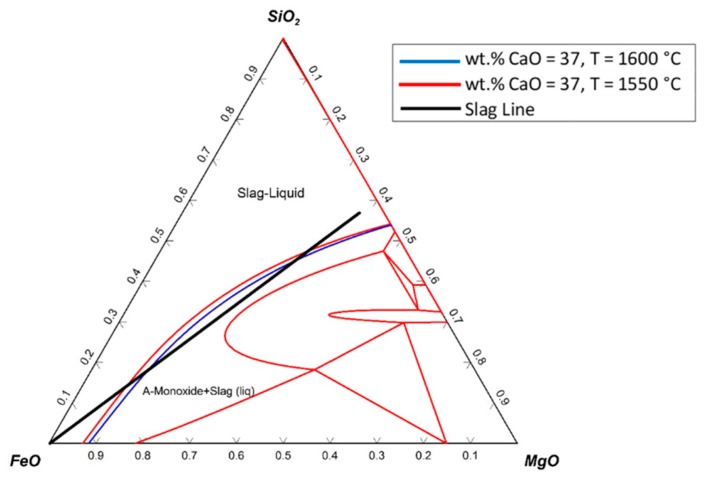
Ternary system of SiO_2_–FeO–MgO with addition of 37 wt.% CaO at 1550 °C and 1600 °C.

The ternary system of SiO_2_–FeO–MgO with an addition of 37 wt.% CaO is shown in Figure 20.

The ternary phase diagram was drawn at 1550 °C, and only one line (blue line) was added to the diagram, which was the liquidus line of the diagram at 1600 °C. The slag line crossed the liquidus line, which implied that during the reduction process, solid particles existed in the slag. Therefore, to melt the slag completely, the temperature had to be increased.

**Figure 21:**
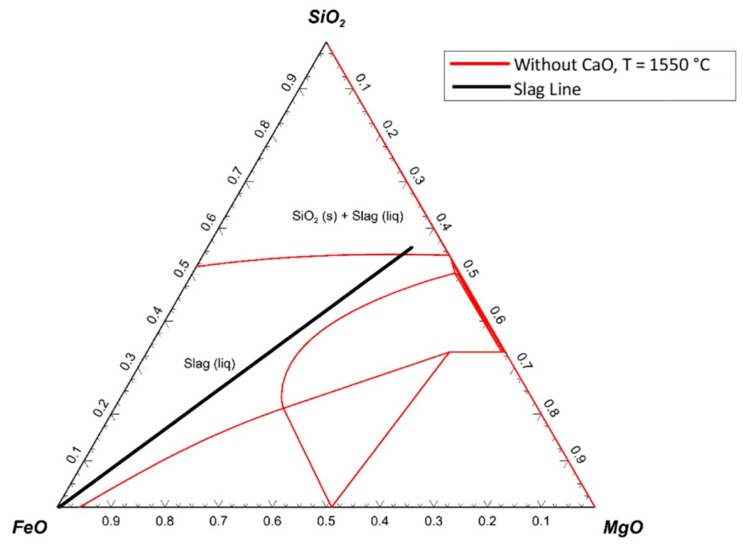
Ternary system of SiO_2_–FeO–MgO with no addition of CaO at 1550 °C.

Furthermore, the SiO_2_–FeO–MgO system with no addition of CaO is shown in Figure 21.

In this system, the slag line was located in the liquid slag. However, with a decrease in the FeO content to less than below 10%, the solid particles of SiO_2_ might be formed.

With a comparison of these three ternary diagrams, it can be suggested that the SiO_2_–FeO–Al_2_O_3_ system with the addition of CaO was appropriate for the operations, as in this system, there was no solid component in the slag phase at 1550 °C. Furthermore, preventing the entrance of refractory materials to the melt can improve the reduction process. One solution is the use of high-alumina refractories such as spinel- instead of magnesia-based refractories.

## 4. Conclusions

Hydrogen can be considered to be a reducing agent for the reduction of iron oxides using the hydrogen plasma smelting reduction process. The main conclusions of the present study are as follows:

- HPSR produces a small amount of CO_2_ due to the contribution of carbon, from graphite electrode, to the reduction reactions. CO_2_ emissions were much lower than those of other iron and steel-making routes.
- $\eta_{H_{2}}$ was high at the beginning of the reduction process, and then, it decreased due to a decrease in the iron oxide content in the liquid slag.
- During the continuous feeding process of iron ore, the $\eta_{H_{2}}$ value could be kept constant. Therefore, we expect to achieve a high $\eta_{H_{2}}$ value in an industrial scale of this process.
- The average $\eta_{H_{2}}$ value was approximately 28% for the samples with a basicity of 1.6 and 2.3.
- Iron oxide, buried by the partially melted material in the steel crucible, could not be reduced. Therefore, the achievement of a high R_D_ value was not possible in the laboratory-scale facility.
- The chemical composition of the slags showed that the FeO content was low; therefore, it seems to be possible to achieve higher degrees of reduction if all the material in the reactor is melted.

## Figures and Tables

**Table 1:** Experimental programme.

Set of Experiments	Experiment	Lime (g)/Iron Ore (g) Ratio	B_2_ (CaO/SiO_2_)	Total Gas Flow Rate (NL/min), 1 atm, 25 °C	H_2_/Ar Ratio (molar%)
Basicity	Ex. 1	0/100	0.0	5	50/50
	Ex. 2	1.7/98.3	0.8		
	Ex. 3	3.3/96.7	1.6		
	Ex. 4	4.9/95.1	2.3		
	Ex. 5	6.4/93.6	2.9		
Continuous feeding	Ex. 6	3.3/96.7	1.6		

**Table 2:** Chemical composition of the iron ore [33].

No.	Element	(wt.%)
1	Fe(III) oxide	92.83
2	Fe(II) oxide	1.07
3	Total Fe	65.81
4	Silica	1.694
5	Aluminium oxide	1.01
6	Manganese (II) oxide	0.22
7	Manganese	0.17
8	Calcium oxide	0.01
9	Magnesium oxide	0.01
10	Phosphorus (V) oxide	0.131
11	Phosphorus	0.057
12	Sodium oxide	0.019
13	Carbon	0.098
14	Zinc	0.004
15	Sulphur trioxide	0.035
16	Total sulphur	0.014
17	Potassium oxide	0.017
18	LOI ^1^	2.79

^1^ Loss of ignition.

**Table 3:** Chemical composition of the calcined lime.

No.	Element	(wt.%)
1	CaO	86.41
2	SiO_2_	4.76
3	MgO	1.05
4	Al_2_O_3_	1.87
5	Fe_2_O_3_	2.50
6	P_2_O_5_	0.11
7	C	0.11
8	S	0.02
9	LOI	3.17

**Table 4:** Grain size distribution of Carajas iron ore and lime [33].

Mesh Size (μm)	Fraction (wt.%)	Cumulative (wt.%)
63–125	34	34
25–63	60	94
0–25	6	100

**Table 5:** Chemical composition of ignition pin and steel crucible [33].

Element	Unit	C	Si	Mn	P	S	Cr	Mo	Ni	Al	Cu
Steel crucible	(wt.%)	0.178	0.261	1.325	0.009	0.005	0.083	0.031	0.168	0.027	0.179
Ignition pin	(wt.%)	0.441	0.217	0.85	0.008	0.028	0.985	0.162	0.085	0.021	0.116

**Table 6:** Chemical composition of produced iron in Ex. 3.

Element	Fe	C	Si	Mn	P	S	Cr	Mo	Ni	Cu
Concentration (wt.%)	99.74	0.003	< 0.001	0.057	0.011	0.03	0.028	0.031	0.045	0.055

**Table 7:** Amount of carbon (g) contributed to the reduction reactions.

Source of Carbon	Unit	Ex. 1	Ex. 2	Ex. 3	Ex. 4	Ex. 5	Ex. 6
HGE	(g)	3.10	3.30	2.70	3.00	2.00	6.4
Ignition pin	(g)	0.066	0.066	0.066	0.066	0.066	0.066
Steel crucible	(g)	0.76	0.19	0.057	0.133	0.513	0.57

**Table 8:** Products and co-products of the experiments in 1495 s of operation.

Parameter	Unit	Ex. 1	Ex. 2	Ex. 3	Ex. 4	Ex. 5	Ex. 6
Produced iron	(g)	26.01	21.63	25.55	28.84	21.67	30.34
M_D_	(%)	39.49	33.41	40.12	46.03	35.14	47.64
Slag weight	(g)	54.73	60.54	55.53	51.45	60.88	49.32
R_D,H2_	(%)	36.70	35.00	40.2	42.00	39.00	34.86
R_D,C_	(%)	21.3	18.78	18.3	20.54	15.98	28.8
R_D,H2+C_	(%)	58.00	53.78	58.5	62.54	54.98	63.66
Average degree of hydrogen utilisation $\left(\bar{\eta_{H_{2}}}\right)$	(%)	26.35	24.71	27.92	28.69	26.22	24.23

**Table 9:** Products and co-products of the experiments in 1975 s of operation.

Parameter	Unit	Ex. 1	Ex. 2	Ex. 4	Ex. 5	Ex. 6
Produced iron	(g)	33.02	28.15	38.05	26.83	43.88
M_D_	(wt.%)	50.14	43.47	60.73	43.51	68.91
Slag weight	(g)	45.64	52.09	39.51	54.18	31.82
R_D,H2_	(%)	42.80	40.80	50.90	43.90	43.57
R_D,C_	(%)	22.59	19.96	21.84	16.89	34.85
R_D,H2+C_	(%)	65.39	60.76	72.74	60.79	78.42
$\bar{\eta_{H_{2}}}$	(%)	22.52	21.11	25.48	21.62	22.18

**Table 10:** Calculated chemical composition of slag for Ex. 5.

Element	Unit	Ex. 5
CaO	(wt.%)	10.04
SiO_2_	(wt.%)	3.444
Al_2_O_3_	(wt.%)	1.94
FeO	(wt.%)	80.62
MgO	(wt.%)	0.14
Fe_2_O_3_	(wt.%)	3.21
K_2_O	(wt.%)	0.03
MnO	(wt.%)	0.34
P	(wt.%)	0.22
S	(wt.%)	0.01

**Table 11:** Composition of the slag measured by EDX.

Parameter	Spectrum	Figure	Unit	O	Mg	Al	Si	Ca	Mn	Fe
Slag composition	1 and 2	15	(wt.%)	41.5	1.3	0.4	16.0	35.2	2.6	3.0
Iron oxide particles	3 and 4	15	(wt.%)	35.5	0.8	0.3	4.3	9.5	1.3	48.3
Slag composition	1, 2 and 3	16	(wt.%)	37.5	4.4	0.9	15.3	36.3	4.8	0.8
Droplets of produced iron	1 and 2	17	(wt.%)	4.8	0.9	0.4	1.3	1.6	1.0	90.0

**Table 12:** Chemical composition of Ex. 5 slag by means of XRF.

Elements	Unit	Ex. 5
CaO	(wt.%)	36.64
SiO_2_	(wt.%)	22.88
Al_2_O_3_	(wt.%)	9.51
FeO	(wt.%)	6.34
MgO	(wt.%)	14.88
MnO	(wt.%)	7.85
TiO_2_	(wt.%)	0.32
Cr_2_O_3_	(wt.%)	0.26
ZrO_2_	(wt.%)	1.17
P_2_O_5_	(wt.%)	0.13
